# Validating the Global Surgery Geographical Accessibility Indicator: Differences in Modeled Versus Patient-Reported Travel Times

**DOI:** 10.1007/s00268-020-05480-8

**Published:** 2020-04-09

**Authors:** Niclas Rudolfson, Magdalena Gruendl, Theoneste Nkurunziza, Frederick Kateera, Kristin Sonderman, Edison Nihiwacu, Bahati Ramadhan, Robert Riviello, Bethany Hedt-Gauthier

**Affiliations:** 1 Program in Global Surgery and Social Change, Department of Global Health and Social Medicine, Harvard Medical School, Boston, USA; 2 World Health Organization Collaborating Center for Surgery and Public Health, Department of Clinical Sciences Lund, Lund University, Lund, Sweden; 3 Department of Epidemiology, Technical University Munich, Munich, Germany; 4 Partners In Health/Inshuti Mu Buzima, Kigali, Rwanda; 5 Center for Surgery and Public Health, Brigham and Women’s Hospital, Boston, USA

## Abstract

**Background:**

Since long travel times to reach health facilities are associated with worse outcomes, geographic accessibility is one of the six core global surgery indicators; this corresponds to the second of the “Three Delays Framework,” namely “delay in reaching a health facility.” Most attempts to estimate this indicator have been based on geographical information systems (GIS) algorithms. The aim of our study was to compare GIS derived estimates to self-reported travel times for patients traveling to a district hospital in rural Rwanda for emergency obstetric care.

**Methods:**

Our study includes 664 women who traveled to undergo a Cesarean delivery in Kirehe, Rwanda. We compared self-reported travel time from home to the hospital (excluding waiting time) with GIS estimated travel times, which were computed using the World Health Organization tool AccessMod, using linear regression.

**Results:**

The majority of patients used multiple modes of transportation (walking = 48.5%, public transport = 74.2%, private transport = 2.9%, and ambulance 70.6%). Self-reported times were longer than GIS estimates by a factor of 1.49 (95% CI 1.40–1.57). Concordance was higher when the GIS model took into account that all patients in Rwanda are referred via their health center (*β* = 1.12; 95% CI 1.05–1.18).

**Conclusions:**

To our knowledge, in this largest to date GIS validation study for geographical access to healthcare in low- and middle-income countries, a standard GIS model was found to significantly underestimate real travel time, which likely is in part because it does not model the actual route patients are travelling. Therefore, previous studies of 2-h access to surgery will need to be interpreted with caution, and future studies should take local travelling conditions into account.

**Electronic supplementary material:**

The online version of this article (10.1007/s00268-020-05480-8) contains supplementary material, which is available to authorized users.

## Introduction

Surgical conditions account for approximately 30% of the global burden of disease, yet 5 billion people lack access to safe, affordable, and timely surgical and anesthesia care [1]. In 2015, The Lancet Commission on Global Surgery recommended six key indicators to assess and track progress of access to surgical services and outcomes. These core indicators measure provider density, operative volume, surgical safety, and financial and geographical access [1].

This last indicator—geographical access—was defined by the Lancet Commission as the percentage of the population who can access, within 2 h, a facility capable of performing the three so-called bellwether procedures: Cesarean section (C section), laparotomy, and open fracture repair [2]. The 2-h cutoff point was chosen from its previously known marker as the critical time from postpartum hemorrhage to death if no intervention is provided [3]. Further, long travel times to reach surgical care including C sections are associated with worse outcomes [4–7]. Therefore, understanding gaps of access within a certain time frame to a facility would allow governments to have an evidence-based method for placement of surgical facilities and staff. This specifically addresses the second delay of the “Three Delays” framework, which outlines three time intervals before treatment is started, [1] delay in seeking care; [2] delay in reaching a health facility; and [3] delay in receiving care [8].

A challenge with the geographical access indicator has been finding high quality, systematic ways to measure it. The gold standard for reporting geographical access is measuring the actual time it takes for patients to travel to the nearest surgically capable hospital. This obviously requires extensive primary data collection, which is both cumbersome, and highly resource intensive, and therefore a significant barrier in low- and middle-income countries. Additionally, it risks missing those patients who needed surgical care but could not reach a hospital due to travel barriers. For this reason, geographic information system (GIS) models, which simulate travel along the road network of a country, have been the primary methodology used to quantify geographical access [9–16], and the results of such studies inform national health planning policy [17–19].

Two large studies in sub-Saharan Africa, which used GIS to model the access to emergency care [16] and to timely and essential surgical care [12], estimated that 71% and 92.5% of the population reside in areas within 2 h of a major hospital catchment, respectively. However, concerns have been raised that commonly employed GIS models underestimate actual travel times in low- and middle-income countries [9]. While GIS may accurately estimate patient travel times in high-income countries [20, 21], there is very limited data on validity of these models in low- and middle-income countries. Given this, the aim of our study was to compare GIS estimates to patient-reported travel times for patients travelling to a district hospital in rural Rwanda for emergency obstetric care.

## Methods

### Study setting

This study was conducted at Kirehe District Hospital (KDH), located in the Eastern Province, Rwanda. KDH—managed by the Rwandan Ministry of Health with support from Partners In Health/Inshuti Mu Buzima (PIH/IMB)—serves a catchment population of nearly 340,000 residents [22]. In Kirehe District, basic outpatient primary care is provided at 16 health centers, from which patients can be transferred to KDH for medical problems requiring hospital care. KDH provides basic secondary level care, including some minor surgical procedures and Cesarean deliveries. Patients needing more complex care are referred to tertiary facilities in Kigali, approximately 3 h away.

In Rwanda, 91% of women deliver in health facilities [23]. The majority of laboring women first seek care at their assigned health center. In cases of emergency, she is then transferred to the district hospital, often by ambulance, where a C section can be performed if needed.

### Study sample, data sources, and data collection

All female patients 18 years or older, who were residents of Kirehe District and delivered via C section at KDH between June 2017 and January 2018 were eligible for inclusion.

Data collectors interviewed patients prior to discharge from the hospital to collect baseline demographic and economic data. Data were collected using REDCap [24], a secure, Web-based application designed to support data capture for research studies in areas with low connectivity, using Android tablets. The following data was gathered on study participants: the name of their home village, whether the patient went to a health center before going to the hospital, the mode of transport from their home to the health center and from the health center to the hospital, the duration of each leg of the journey, the wait time at the health center or hospital admission area, and the cost of the trip.

Study staff informed patients about the study and obtained written consent. Approvals were received from the Partners In Health/Inshuti Mu Buzima (PIH/IMB) Research Committee and the Rwandan National Health Research Committee, and ethical approvals from the Rwanda National Ethics Committee (Kigali, Rwanda; no. 848/RNEC/2016) and Partners Human Research Committee (Boston, Massachusetts, USA; no. 2016P001943/MGH). The study was approved by the Rwandan Ministry of Health before the start of data collection.

### GIS methodology

We reconciled patient-reported village names with official location names from the National Institute of Statistics Rwanda [25]. For each of the 612 villages in Kirehe District, we calculated the geographical centroid. Patients matched to that village were assumed to be starting their journey at this central location. The geographic boundaries of Rwandan villages were obtained from the Global Administrative Areas database [26].

GIS estimated travel times were computed using the WHO tool AccessMod, software version 5.0 [27]. AccessMod calculates the shortest possible travel time from every point in the analyzed region, taking travel speed into account. The region is discretized into cells, which are assigned a travel speed. The analysis was performed with a cell size of roughly 100 m. In order to emulate previous GIS studies [9, 13–16], roads were classified into primary, secondary, and tertiary roads, and the travel speed was assumed to be 100, 50, and 30 km/h, respectively. All remaining cells were set to a speed of 5 km/h (approximate walking speed), apart from those representing rivers or bodies of water, which were set as non-traversable. Data on the Rwandan road network, rivers, and bodies of water were obtained from OpenStreetMaps [28].

Two scenarios were calculated. In the first, patients were assumed to travel the most direct route possible from home to Kirehe District Hospital. We refer to this model as the “standard model” as this is the pathway patients are assumed to take in most studies that utilize GIS methodology. In the second, patients were assumed to first travel to their assigned health center, and then from the health center to the hospital, as this is the prescribed referral pattern in the Rwanda public health sector.

### Statistical analysis

In our analyses, we compared patient-reported travel times to GIS estimated travel times. For patient-reported travel time, we only included time in transit (time from home to health center and health center to hospital) and did not include patient wait times at the health center or hospital. This method was chosen because it is most comparable to the GIS estimated travel times which would also not include any delays in the estimates.

We used univariable linear regression to compare patient-reported and GIS estimated travel times. We did not include an intercept in the regression specification. Maps were produced to illustrate patient-reported and GIS estimated travel times, using the raw output of the AccessMod tool and an interpolated surface of patient-reported travel times. The interpolation was produced using inverse distance weighting. All analyses were performed in R (version 3.4.1, R Foundation for Statistical Computation, Vienna, Austria).

## Results

### Demographics

A total of 664 women who underwent a C section at Kirehe District Hospital were included in the study. The location of the home village of included patients is displayed in Figure S1. We excluded three patients from analysis because their data were outliers deemed to be likely caused by data entry errors. The median age was 26 years (interquartile range (IQR): 23, 31 years), most had primary education (470 patients, 70.8%), and a monthly household monetary income of less than 10,000 Rwandan francs (approximately USD $12, 518 patients, 78.0%) (Table 1). The most common mode of transportation from home to the health center was public transport (477 patients, 71.8%) and walking (183 patients, 27.6%), with only a small fraction of patients reporting private transport or ambulance. Conversely, the most common form of transport from the health center to the hospital was the use of an ambulance (467 patients, 70.3%) and walking (164 patients, 24.4%). All patients who reported walking to the hospital came from the nearby Kirehe Health Center.

**Table 1:** Demographics of the study population

Variable	*n* (%)
*n*	664
Age [median (IQR)]	26 [23, 31]
Education level	
No education	59 (8.9)
Primary education	470 (70.8)
Secondary or higher education	135 (20.3)
Household monthly income	
0–10,000 Rwf	518 (78.0)
10,000–20,000 Rwf	69 (10.4)
20,000–30,000 Rwf	26 (3.9)
>30,000 Rwf	51 (7.7)
Modes of transportation used from home to health center^a^	
Walking	183 (27.6)
Public	477 (71.8)
Private	12 (1.8)
Ambulance	8 (1.2)
Modes of transportation used from health center to hospital^a^	
Walking	162 (24.4)
Public	36 (5.4)
Private	9 (1.4)
Ambulance	467 (70.3)

^a^Multiple answers were allowed

### Travel time

**Fig. 1:**
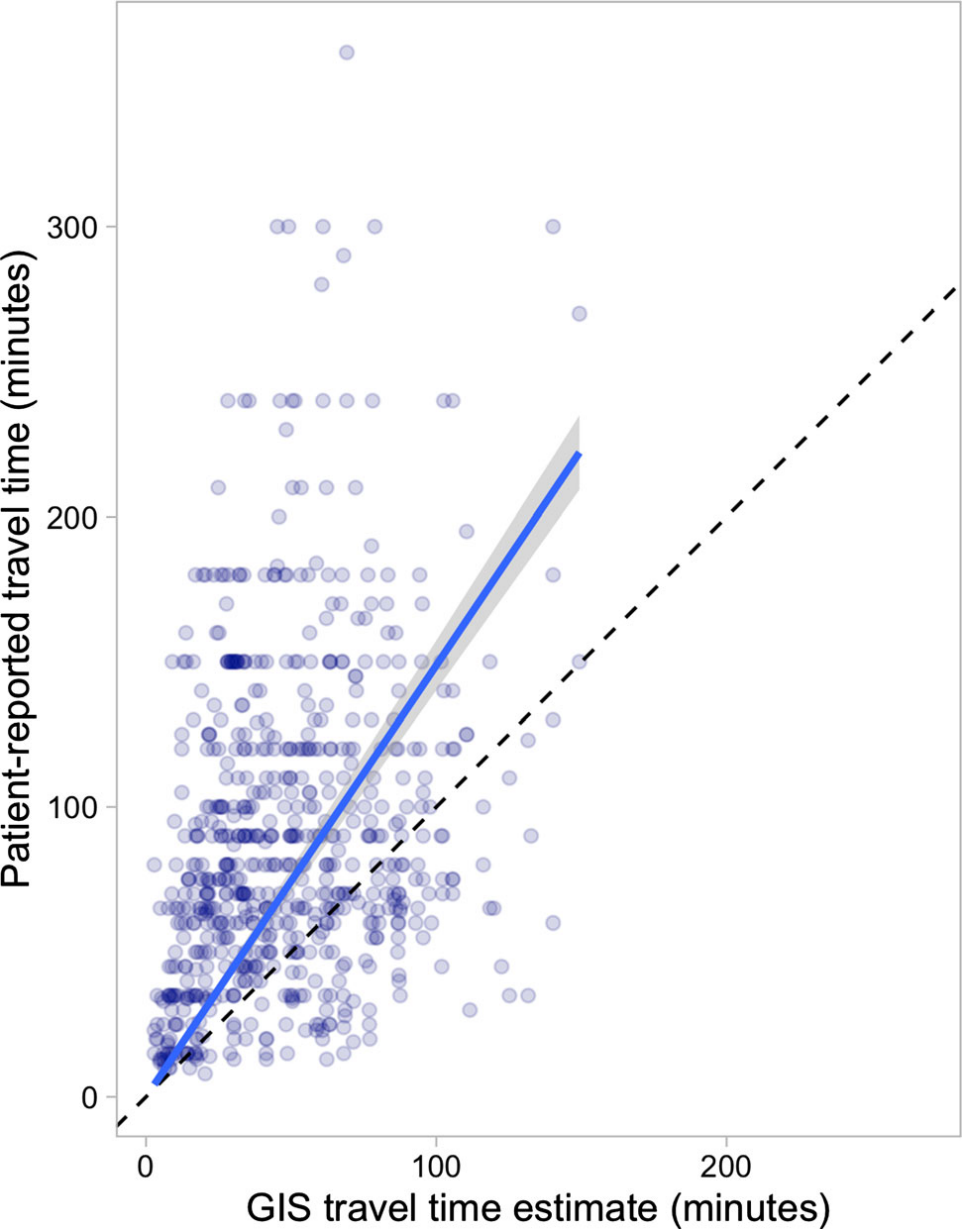
Relationship between patient-reported and GIS estimated travel times in the standard GIS model. The dashed line represents equality between the two estimates, and the solid line linear regression

**Fig. 2:**
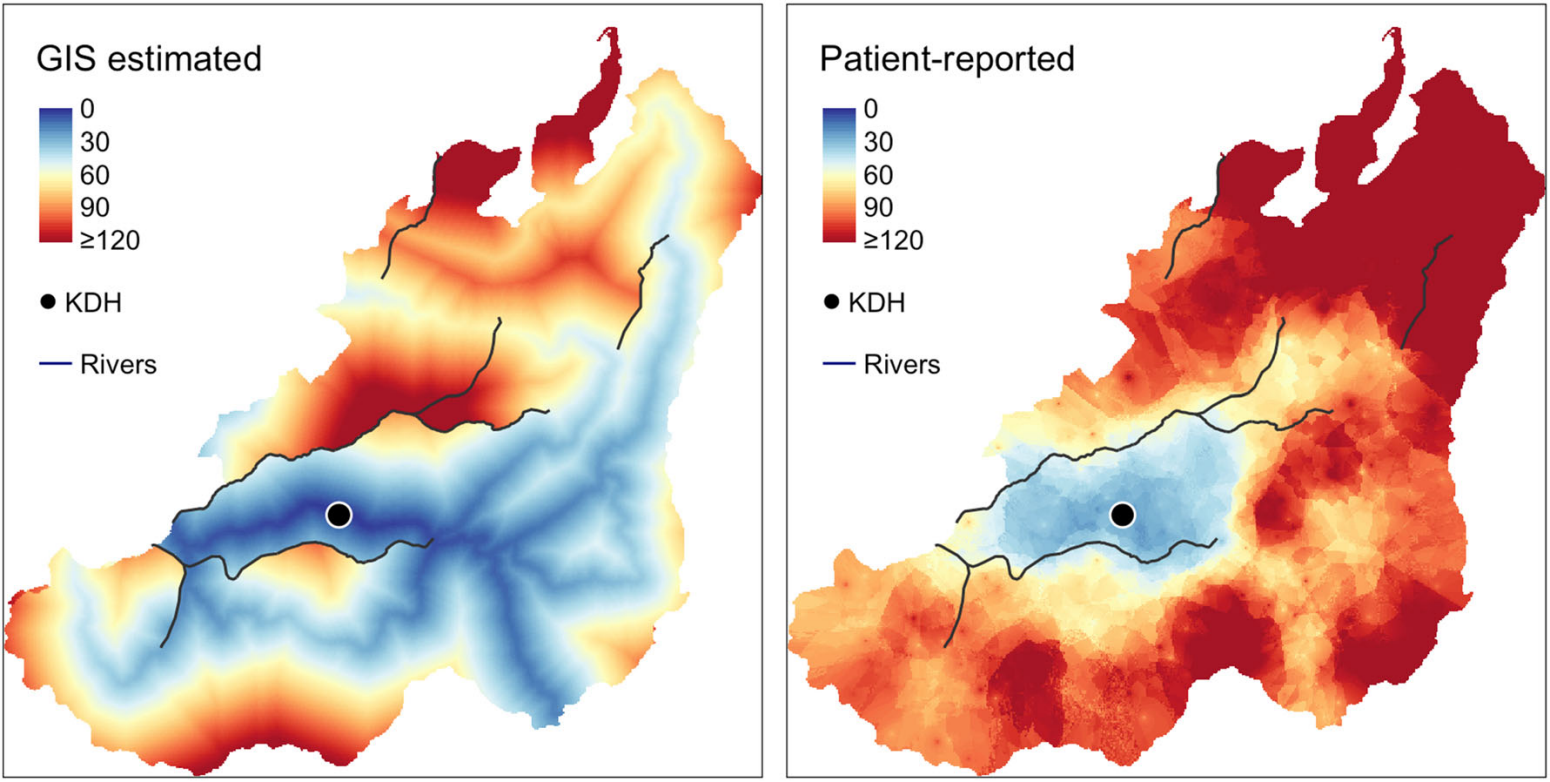
Map comparison of GIS estimated and patient-reported travel times, time in minutes from home to the Kirehe District Hospital

The total transport time reported by patients, not including waiting at the health center, was longer than the time estimated by the standard AccessMod estimate (mean 88.3 and 47.7 min, respectively). In the linear regression analysis, the patient-reported estimate was 1.5 times greater than the AccessMod estimate [*β* = 1.49, 95% confidence interval (CI) 1.40, 1.57] (Figs. 1 and 2).

**Fig. 3:**
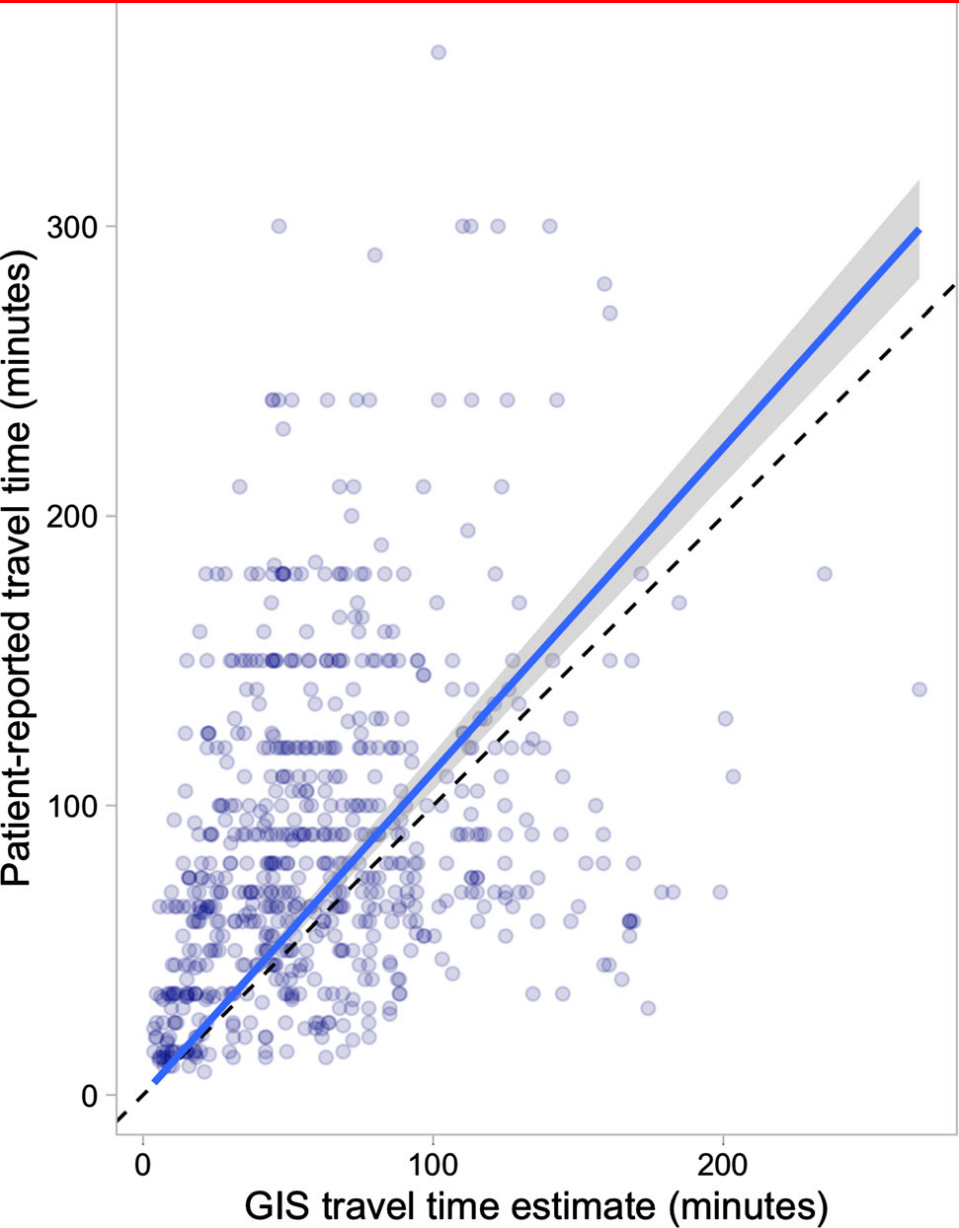
Relationship between patient-reported and GIS estimated travel times when accounting for journeying via a Health Center in the GIS model. The dashed line represents equality, and the solid line linear regression

**Fig. 4:**
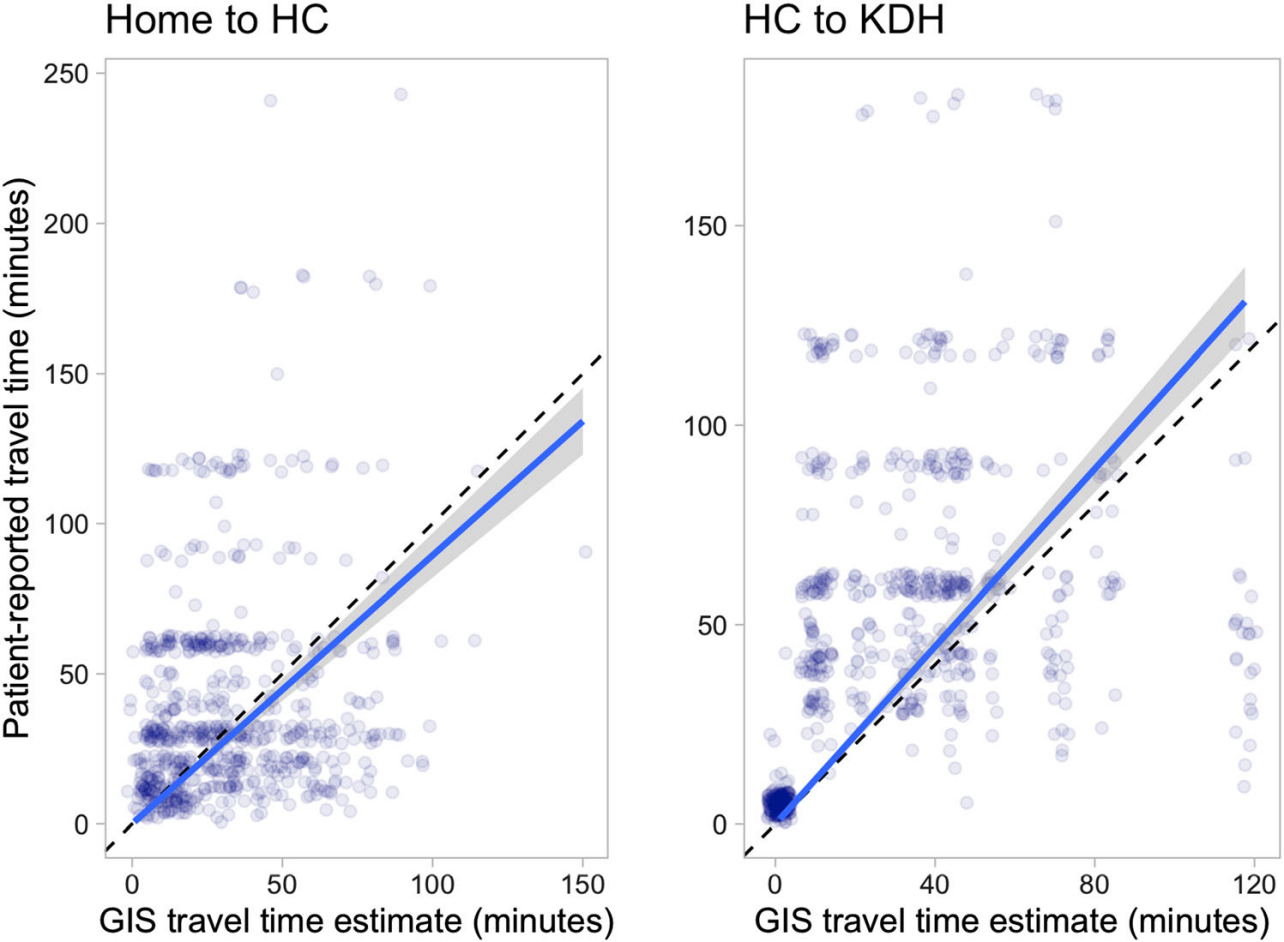
Relationship between patient-reported and GIS estimated travel times, from home to the health center (left) and from the health center to Kirehe District Hospital (right). The dashed line represents equality, and the solid line linear regression. *HC* Health Center, *KDH* Kirehe District Hospital

For the estimates that accounted for journeying via the assigned health center, the total AccessMod estimates were closer to travel times reported by patients (mean 62.3 min, *β* = 1.12; 95% CI 1.05, 1.18) (Fig. 3). The AccessMod slightly underestimated the patient-reported travel time for the home-to-health center leg, (*β* = 0.89; 95% CI 0.82, 0.97) and overestimated the patient-reported time from the health center to the hospital (*β* = 1.11; 95% CI 1.04, 1.19] (Fig. 4).

## Discussion

At a time when there is a global interest and movement in expanding surgical care in low- and middle-income countries, it is imperative that an accurate tool is accepted as a way to measure geographic access. GIS has readily been used in high-income countries to measure just this, but the utility in LMICs has largely been unknown. Our study found that the standard approach to estimate geographical accessibility underestimates the true patient experience, as the GIS estimated travel times were significantly lower than those reported by patients. Adjusting the model to account for the fact that patients access hospital care via the health center results in estimates considerably closer to the patient-reported travel time, although it should be noted that wait times to secure an ambulance at the health center to travel to the hospital were not included.

In high-income countries, validation studies have shown GIS to be a relatively accurate estimator of patient travel times in both elective and emergency cases. For example, a study of 475 cancer patients in the North of England demonstrated that 90% of travel time estimates were within 15 min, [20] and a study by Patel et al. [21] that looked at the ground ambulance pre-hospital times for emergency adult patient trips within the Calgary area, Canada found that GIS estimates were slightly underestimating real travel time. In LMICs, three small studies from Uganda, Ghana, and Afghanistan [29–31] have compared GIS estimates to patient-reported travel times, although none have used travel speed assumptions similar to those used in the global surgery literature. To our knowledge, ours is the largest to date validation of GIS modeling travel time in an LMIC and the first study to use AccessMod.

The results presented in this study pose important implications for further studies of the geographical access to surgery and emergency care. We found a standard GIS model to systematically underestimate travel time. There are several potential reasons for this discrepancy, including assumptions about travel speeds, modes of transport, and travel routes. Previous studies of 2-h access will need to be interpreted with caution, and in light of the local context. Decision makers will need to take this into account when planning the scale-up of surgical capacity, and it seems likely that previous estimates stating that 71–92% of the sub-Saharan population is able to reach emergency care within 2 h [13, 16] is overly optimistic. If patients cannot reach hospital care within 2 h when travelling via a health center, then policies requiring such stepwise referrals may need to be reconsidered. At the very least, in countries where this is the case, this additional delay will need to be accounted for in planning of infrastructure and deploying new capacity for surgical services to existing facilities. It should also be noted GIS only models one delay in reaching care, travelling to the hospital. Table 2 outlines the three delay framework, how it relates to GIS modeling, and some potential sources of error in GIS modeling.

**Table 2:** Assumptions of GIS calculations and the three delay framework, including factors which complicate modeling and examples of delays which current models generally do not account for

Assumption	Three Delay framework	Potential difficulties in measurement	Example of unaccounted delay
Patients will decide to seek care directly when need arises	First delay	Patient and disease specific	Securing funds for travel and/or care
Patients can start their travel right away	Second delay	Highly context and patient specific	Waiting for transport, e.g., ambulance or private
Patients can travel at declared speed	Second delay	Context specific	Poor road conditions, using slower modes of transport
Patients choose the fastest route	Second delay	Depends on setting, referral system	Travels another route, e.g., via lower tier hospital
Upon arrival, there is capacity to take care of patient	Third delay	Costly, may vary depending on time of day	No surgeon on site, overfilled ER

Note that GIS is used to quantify the second delay

The results of this study need to be interpreted in light of some limitations. Most importantly, the study only includes one district in Rwanda, and it is possible that GIS models would perform better or worse in different conditions based on infrastructure conditions, geographic topology, and various other factors. However, most of sub-Saharan Africa does require the health center-to-hospital referral for surgical care and we posit that the failure to account for this in model estimates will result in systematic underestimates even if the exact parameters are not generalizable. We note that we only modeled one set of travelling speed assumptions, chosen due to its predominance in the literature [9, 13–16] but that in theory a Rwanda-specific set of speeds could be generated and could yield more accurate results. Further, self-reported travel time may contain recall bias or rounding errors, but were collected within days of the trip.

To the best of our knowledge, this is the largest study to date comparing GIS modeling to real-world data in a low- and middle-income country and the first using a standard method for generating data for the geographic access for surgery indicator. While we found a high degree of correlation between travel times as estimated by our GIS model and reported by patients, GIS estimates were systematically lower. Changing the GIS model to take the health center detour into account significantly improved the concordance of modeled and patient-reported results. More research will be needed to further understand the transport conditions in varying contexts, and future GIS modeling studies on geographical access should take those local conditions into account.

## Electronic supplementary material

Below is the link to the electronic supplementary material.

Figure S1 Map of home village of study participants. The left panel shows Rwanda, with Kirehe district in blue. Right panel shows Kirehe District, where point size represents the number of included patients in each village. (PNG 158 kb)
