# Supplementary figures and images for: Validating the Global Surgery Geographical Accessibility Indicator: Differences in Modeled Versus Patient-Reported Travel Times
Source: World J Surg. 2020 Apr 9;44(7):2123–30. doi: 10.1007/s00268-020-05480-8 (PMC7266844; doi:10.1007/s00268-020-05480-8)

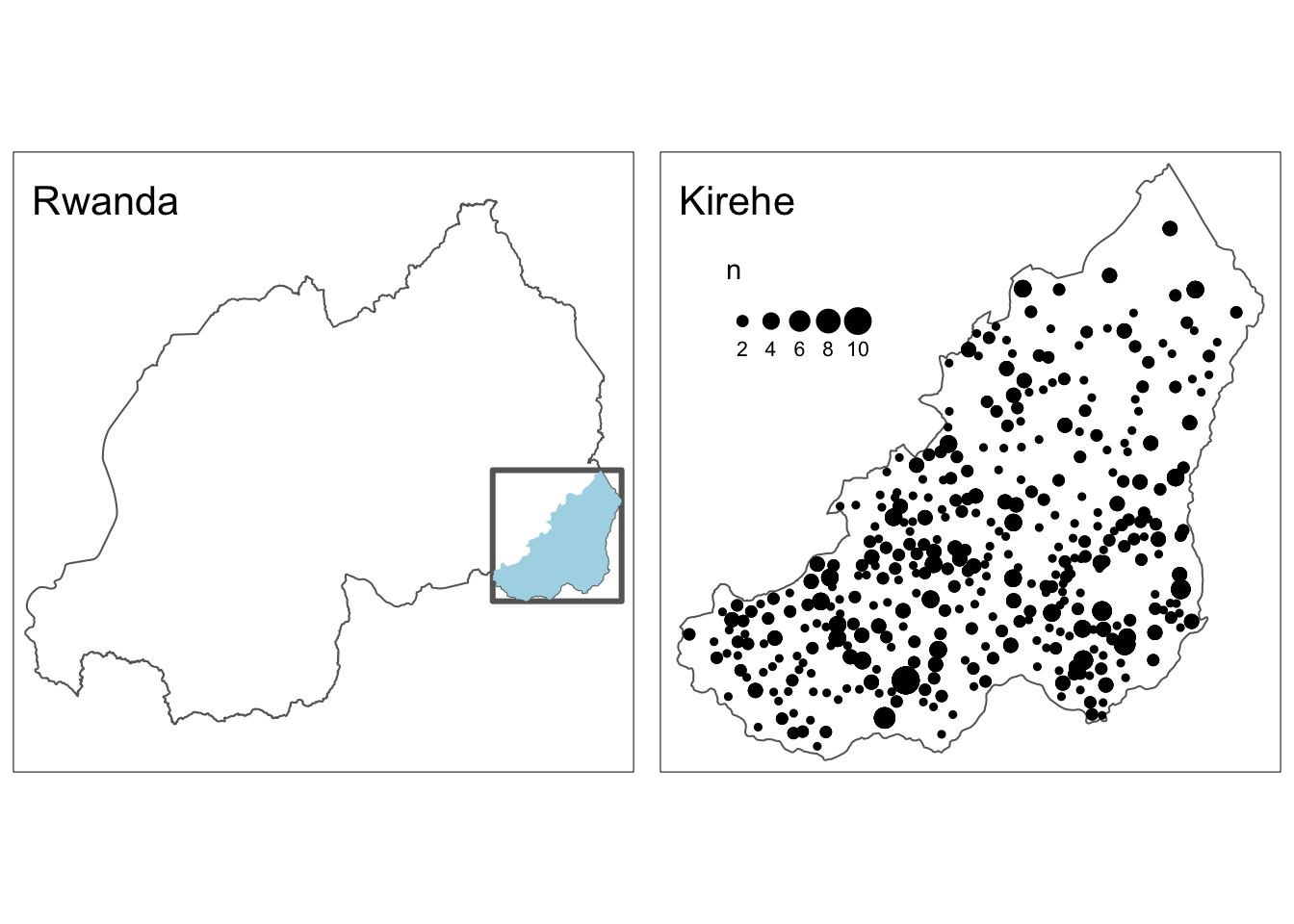

Supplement: Supplementary file 1 — Map of home village of study participants. The left panel shows Rwanda, with Kirehe district in blue. Right panel shows Kirehe District, where point size represents the number of included patients in each village. (PNG 158 kb) [file 268_2020_5480_MOESM1_ESM.png]
